# Fas Ligand DNA Enhances a Vaccination Effect by Coadministered DNA Encoding a Tumor Antigen through Augmenting Production of Antibody against the Tumor Antigen

**DOI:** 10.1155/2015/743828

**Published:** 2015-02-18

**Authors:** Boya Zhong, Guangyu Ma, Ayako Sato, Osamu Shimozato, Hongdan Liu, Quanhai Li, Masato Shingyoji, Yuji Tada, Koichiro Tatsumi, Hideaki Shimada, Kenzo Hiroshima, Masatoshi Tagawa

**Affiliations:** ^1^Department of Hematology, Fourth Hospital of Hebei Medical University, Shijiazhuang 050035, China; ^2^Division of Pathology and Cell Therapy, Chiba Cancer Center Research Institute, Chiba 260-8717, Japan; ^3^Department of Molecular Biology and Oncology, Graduate School of Medicine, Chiba University, Chiba 260-8670, Japan; ^4^Laboratory of DNA Damage Signaling, Chiba Cancer Center Research Institute, Chiba 260-8717, Japan; ^5^Department of Immunology, Hebei Medical University, Shijiazhuang 050017, China; ^6^Cell Therapy Center, 1st Hospital of Hebei Medical University, Shijiazhuang 050031, China; ^7^Department of Thoracic Diseases, Chiba Cancer Center, Chiba 260-8717, Japan; ^8^Department of Respirology, Graduate School of Medicine, Chiba University, Chiba 260-8670, Japan; ^9^Department of Surgery, School of Medicine, Toho University, Tokyo 143-8541, Japan; ^10^Department of Pathology, Tokyo Women's Medical University Yachiyo Medical Center, Yachiyo 276-8524, Japan

## Abstract

Interaction of Fas and Fas ligand (FasL) plays an important role in the regulation of immune responses by inducing apoptosis of activated cells; however, a possible role of FasL in DNA vaccination has not been well understood. We examined whether administration of DNA encoding *FasL* gene enhanced antitumor effects in mice that were vaccinated with DNA expressing a putative tumor antigen gene, *β*-*galactosidase* (*β*-*gal*). Growth of *β*-gal-positive Colon 26 tumors was retarded in the syngeneic mice immunized with *β*-gal and FasL DNA compared with those vaccinated with *β*-gal or FasL DNA. We did not detect increased numbers of *β*-gal-specific CD8^+^ T cells in lymph node of mice that received combination of *β*-gal and FasL DNA, but amounts of anti-*β*-gal antibody increased with the combination but not with *β*-gal or FasL DNA injection alone. Subtype analysis of anti-*β*-gal antibody produced by the combination of *β*-gal and FasL DNA or *β*-gal DNA injection showed that IgG2a amounts were greater in mice injected with both DNA than those with *β*-gal DNA alone, but IgG2b amounts were lower in both DNA-injected than *β*-gal DNA-injected mice. These data suggest that FasL is involved in boosting humoral immunity against a gene product encoded by coinjected DNA and enhances the vaccination effects.

## 1. Introduction

DNA vaccine holds an advantage over conventional types which use a target protein as an immunogen in the stability and its relatively low systemic toxicity and has been examined for the efficacy in experimental animal models and moreover in clinical settings [1, 2]. Previous studies demonstrated that administration of DNA potentially induced immune responses to an antigen encoded by the DNA and produced protective immunity [3, 4]. Nevertheless, the low transduction efficacy with DNA vaccine administered* in vivo* hampered extensive clinical application. A possible use of a molecular adjuvant, which can be also administered as DNA, can circumvent the inefficient transduction level [5, 6].

Fas ligand (FasL), type II transmembrane protein, is a member of tumor necrosis factor family with 40 kDa and plays a major role in inducing programmed cell death when it is interacted with Fas [7]. The Fas/FasL interactions induce apoptosis of immune cells including T, B cells and macrophages and the cell death is often associated with activated stages of immune cells. The activation-induced cell death is a mechanism to inhibit excessive immune responses and to terminate ongoing immunity. Naive T cells come to express FasL upon antigen stimulation, and the activated T cells are subjected to apoptosis, which ceases the T cells-mediated responses [8]. Moreover, expression of FasL contributed to enhanced antigen uptake in dendritic cells [9], which indicates that FasL are involved not only in decreasing immunity but in augmenting immune responses. The Fas/FasL interactions thus regulate immune responses in multiple ways.

In the present study, we examined a role of FasL expression as an adjuvant in vaccination effects on tumor growth. We used *β*-galactosidase (*β*-gal) that was used as a putative tumor antigen in a murine animal model and tested whether administration of FasL DNA modulated antitumor responses induced by immunization of *β*-gal-encoding DNA.

## 2. Materials and Methods

### 2.1. Cells and Mice

Murine colon carcinoma Colon 26 cells and packaging cells, Ψ2 and PA317, were maintained with RPMI1640 or DMEM medium supplemented with 10% fetal calf serum. BALB/c mice were purchased from CLEA Japan SLC (Tokyo, Japan).

### 2.2. Transduction of Tumor Cells

The retrovirus vector LXSN (provided by Dr. A.D. Miller, Fred Hutchinson Cancer Research Center, Seattle, WA, USA) was used to harbor *β*-gal cDNA. The retroviral DNA was transfected into ecotropic Ψ2 cells and the cell-free supernatants were further incubated with amphotropic PA317 cells. The culture supernatants of PA317 cells were used for infecting Colon 26 cells. Transduction of Colon 26 cells with the *β-gal* gene (Colon 26/*β*-gal) was confirmed with 5-bromo-4-chloro-3-indolyl *β*-D-galactoside (X-gal) staining.

### 2.3. DNA Administration and X-Gal Staining

Full-length *β*-gal, mouse FasL cDNAs were cloned into expression plasmid vectors, pcDNA3 (the transgene is activated by cytomegalovirus promoter) or pCAGGS (CAG promoter), respectively, and plasmid DNA of pcDNA3/*β*-gal, pCAGGS/FasL was purified with an endotoxin-free DNA extraction kit (Qiagen, Hilden, Germany). Cardiotoxin (Latoxan, Valence, France) was injected into thigh muscle of mice 5 days before DNA administration. For investigation of *β*-gal expression, DNA (10 *μ*g or 50 *μ*g) was injected in the same area in thigh, and the muscles were fixed with 2% formaldehyde and 0.05% glutaraldehyde and then reacted with X-gal solution [10].

### 2.4. Antitumor Effects Produced by DNA Injection

BALB/c mice were injected with cardiotoxin (1 *μ*mol) and with pcDNA3/*β*-gal and/or pCAGGS/FasL DNA (50 *μ*g each) on day 5. They were subcutaneously inoculated with Colon 26/*β*-gal cells (1 × 10^6^) 21 days after DNA injections, and the tumor volume was calculated according to the formula (1/2 × length × width^2^). All the animal experiments were approved by the Animal Experiment and Welfare Committee at Chiba Cancer Center Research Institute.

### 2.5. Detection of Antigen-Specific T Cell Population

A specific epitope peptide sequence TPHPARIGL of *β*-gal for H-2L^d^ haplotype was loaded onto the soluble dimeric H-2L^d^-linked immunoglobulin (Ig) complex (Dimer X I, BD Bioscience, San Jose, CA, USA) [11]. Inguinal lymph node cells were reacted with fluorescence isothiocyanate- (FITC-) conjugated anti-mouse CD8 antibody (Ab) (BD Bioscience) and with the dimeric H-2L^d^-linked Ig complexes loaded with the peptide, followed by phycoerythrin-conjugated anti-mouse IgG_1_ (BD Bioscience). The dimeric complex-positive or -negative and CD8^+^ T cells were examined with FACSCalibur (BD Bioscience) and CellQuest software (BD Bioscience).

### 2.6. Detection of Anti-*β*-Gal Antibody

Amounts of anti-*β*-gal Ab were estimated with enzyme-linked immunosorbent assay (ELISA) using purified *β*-gal protein (Invitrogen, Carlsbad, CA, USA) as a standard and horseradish peroxidase- (HRP-) conjugated anti-mouse IgG Ab (GE Healthcare, Buckinghamshire, UK) as previously described [12]. An isotype of anti-*β*-gal Ab in mice sera was detected with HRP-conjugated anti-mouse IgG_1_ (SouthernBiotech, Birmingham, AL, USA), IgG_2a_, IgG_2b_, or IgM (Invitrogen) Ab. The values of respective isotypes were calculated based on optical density at 450 nm since isotype-specific standard anti-*β*-gal Ab is currently unavailable.

### 2.7. Statistical Analysis

We conducted statistical analyses with the one-way analysis of variance (ANOVA) and *P* values less than 0.05 were judged as significant.

## 3. Results

### 3.1. Immunization with DNA Encoding *β-Gal* Gene

**Figure 1:**
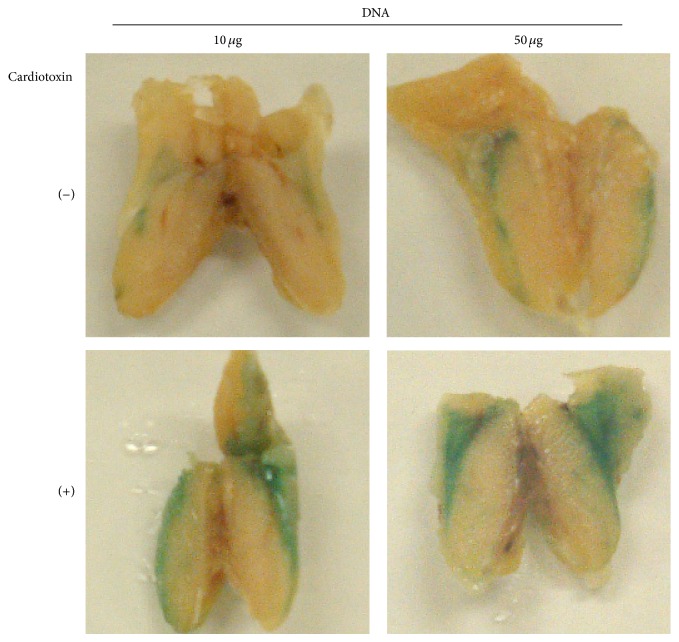
Expression of *β*-gal protein in mice that received DNA immunization. BALB/c mice were injected with or without cardiotoxin at thigh muscles and then with pcDNA3/*β*-gal (10 or 50 *μ*g) at the same muscles 5 days later. The thigh muscles were stained with the X-gal staining 5 days after DNA immunization.

We examined expression of the *β-gal* gene in muscles of mice that were injected with pcDNA3/*β*-gal plasmid DNA and investigated a possible enhancement of the gene expression with a cardiotoxin treatment (Figure 1). Cardiotoxin destroys muscle tissues and the regeneration process facilitates uptake of DNA [13, 14]. Expression levels of *β*-gal detected with the X-gal staining method depended on amounts of pcDNA3/*β*-gal DNA used, and the cardiotoxin treatment prior to DNA administration augmented the *β*-gal expression. We thereby treated mice with cardiotoxin and 5 days later immunized the mice with 50 *μ*g DNA in the following experiments.

### 3.2. Enhanced Antitumor Effects by FasL DNA Immunization

**Figure 2:**
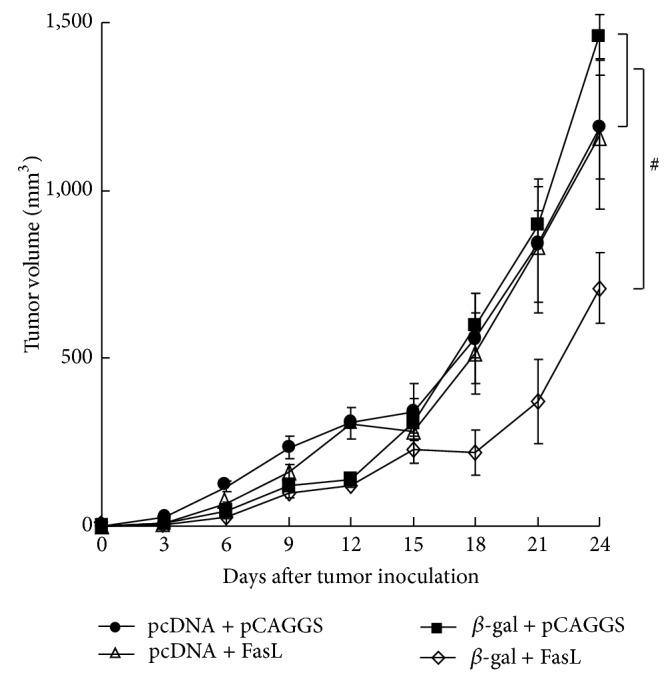
Antitumor effects produced by DNA immunization. BALB/c mice (*n* = 6 or 7) were treated with cardiotoxin and 5 days later with DNA (50 *μ*g for each), pcDNA3 + pCAGGS, pcDNA3 + pCAGGS/FasL (FasL), pcDNA3/*β*-gal (*β*-gal) + pCAGGS, or pcDNA3/*β*-gal + pCAGGS/FasL (*β*-gal + FasL). The mice were then inoculated with Colon 26/*β*-gal cells (1 × 10^6^) 21 days after DNA injections. The tumor growth of mice injected with cDNA3/*β*-gal + pCAGGS/FasL was significantly retarded 21 days after the tumor inoculation compared with that of mice inoculated with pcDNA3 + pCAGGS, pcDNA3 + pCAGGS/FasL, or pcDNA3/*β*-gal + pCAGGS. ^#^ *P* < 0.05.

We investigated whether immunization with DNA encoding a putative tumor antigen achieved antitumor effects. We firstly transduced murine Colon 26 cells with the *β-gal* gene and confirmed that the growth of Colon 26/*β*-gal cells* in vitro* and* in vivo* was not different from parental Colon 26 cells. Syngeneic BALB/c mice were injected with cardiotoxin and then with DNA expressing the *β-gal* and/or* FasL* gene or vector DNA as a control. The mice were then inoculated with Colon 26/*β*-gal cells and the tumor volumes were monitored. Growth of Colon 26/*β*-gal cells was not statistically different among mice that were inoculated with vector DNA, pcDNA3/*β*-gal, or pCAGGS/FasL DNA (Figure 2), and the tumor growth in these mice was not different from that in naive mice (data not shown). In contrast, the tumor growth in mice that received both pcDNA3/*β*-gal and pCAGGS/FasL DNA was retarded compared with that in mice immunized with vector DNA, pcDNA3/*β*-gal, or pCAGGS/FasL DNA (*P* < 0.05). These data indicated that immunization of DNA encoding the *β-gal* or the* FasL* gene alone did not produce antitumor effects but a combinatory use of both DNA achieved vaccination effects.

### 3.3. Constant Frequency of Antigen-Specific T Cells

**Figure 3:**
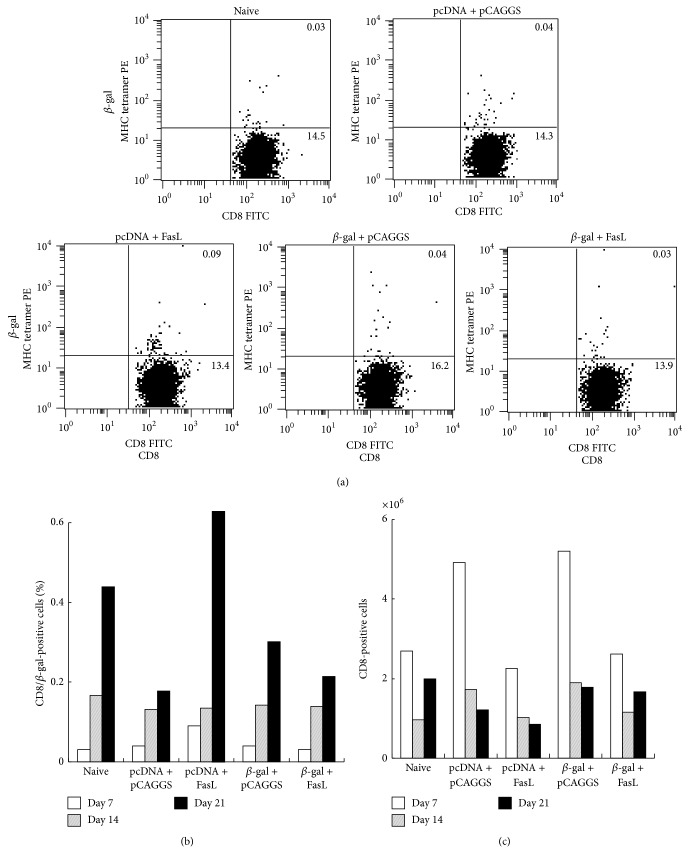
Representative data on frequency of *β*-gal-specific CD8^+^ T cells in inguinal lymph nodes. BALB/c mice were pretreated with cardiotoxin and then uninjected (naive) or injected with pcDNA3 + pCAGGS, pcDNA3 + pCAGGS/FasL, pcDNA3/*β*-gal + pCAGGS, or pcDNA3/*β*-gal + pCAGGS/FasL (50 *μ*g DNA for each). (a) Representative cell surface staining profiles of the lymph nodes 7 days after DNA immunization. The number indicates a percentage of each fraction. (b) Percentages of CD8^+^/*β*-gal-positive T cells in respective mice on days 7, 14, and 21 after DNA immunization. (c) Percentages of CD8^+^ T cells in respective mice on days 7, 14, and 21 after DNA immunization.

We investigated a possible mechanism underlying the antitumor effects produced by the combinatory immunization. We firstly examined induction of antigen-specific CD8^+^ T cells that mediated cytotoxic activities. Cells from inguinal lymph nodes that were obtained on days 7, 14, and 21 after DNA immunization were stained with antibody against CD8 Ab and peptide-loaded class I antigens (Figures 3(a) and 3(b)). Immunization of both *β-gal* and FasL DNA did not increase the antigen-positive CD8^+^ T cells compared with other DNA immunizations or naive cases irrespective of days examined. We also calculated total CD8^+^ cell numbers in lymph nodes and found that the numbers in mice which received both *β-gal* and FasL DNA did not increase compared with those in other experimental groups (Figure 3(c)). These data suggest that cytotoxic T cells were not responsible for the antitumor effects by immunization of *β*-gal and FasL DNA.

### 3.4. Increased Ab against *β*-Gal

**Figure 4:**
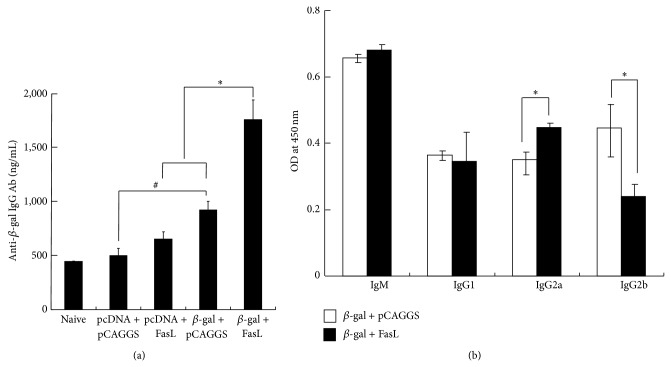
Production of ant-*β*-gal Ab after DNA immunization. BALB/c mice were treated with cardiotoxin followed by DNA immunization, pcDNA3 + pCAGGS, pcDNA3 + pCAGGS/FasL, pcDNA3/*β*-gal + pCAGGS, or pcDNA3/*β*-gal + pCAGGS/FasL (50 *μ*g DNA for each). (a) Concentrations of anti-*β*-gal IgG Ab in the mice 14 days after DNA immunization were measured with an ELISA assay (*n* = 3). (b) Concentrations of Ig subclasses were expressed as an optical density value (*n* = 3). ^#^ *P* < 0.05, ^*^ *P* < 0.01.

We examined a possible involvement of humoral immunity in the antitumor effects by the immunization of *β*-gal and FasL DNA. We firstly measured serum concentrations of anti-*β*-gal IgG Ab produced by DNA immunization (Figure 4(a)). Injection of *β*-gal DNA increased anti-*β*-gal Ab as demonstrated between the group injected with pcDNA3/*β*-gal + pCAGGS DNA and that with pcDNA3 + pCAGGS DNA (*P* < 0.05), whereas injection of FasL DNA did not (pcDNA3 + pCAGGS/FasL versus pcDNA3 + pCAGGS, *P* = 0.48). Coinjected FasL DNA together with *β*-gal DNA however augmented the Ab production since the group injected with pcDNA3/*β*-gal + pCAGGS/FasL DNA showed greater responses than that with pcDNA3 + pCAGGS/FasL or pcDNA3/*β*-gal + pCAGGS DNA (*P* < 0.01). We then further examined a possible influence of FasL DNA injection on differential Ig isotype production (Figure 4(b)). IgG_2a_ amounts were greater in immunization with both *β*-gal and FasL DNA than in that with *β*-gal DNA alone (*P* < 0.01), whereas IgG_2b_ amounts were rather less in the injection of *β*-gal plus FasL DNA than in that of *β*-gal DNA alone (*P* < 0.01). The amounts of IgM and IgG_1_ were not different between the mice injected with both *β*-gal and FasL DNA and those with *β*-gal DNA (IgM; *P* = 0.29, IgG_1_; *P* = 0.85).

## 4. Discussion

The present study demonstrated that administration of FasL DNA functioned as an adjuvant and augmented Ab production against a tumor antigen. The adjuvant effects by FasL expression generated antitumor immunity which was primed by DNA vaccination targeting the tumor antigen. A combinatory use of DNA against the tumor antigen and FasL however did not influence the antigen-positive CD8^+^ T cell numbers, suggesting that the antitumor immunity by DNA vaccine was not attributable to cell-mediated immunity. In contrast, previous studies showed that vaccination of a tumor antigen with plasmid DNA achieved antitumor effects through antigen-positive cytotoxic T cells [15]. Moreover, the Fas/FasL interactions have negative effects on efficacy of DNA vaccine not only by inducing apoptosis of cytotoxic T cells [16] but also by promoting clearance of injected plasmid DNA [17]. Nevertheless, Dharmapuri et al. demonstrated that downregulation of Fas with siRNA did not influence the antitumor responses produced by DNA encoding a tumor antigen although siRNA for Bak1 or caspase-8, both of which were involved in apoptotic processes, enhanced the responses in the same experimental settings [18]. A possible role of FasL and Fas in the context of DNA vaccine* in vivo* is thus subjected to multiple factors such as immunological microenvironments where tumors develop.

The present study did not examine a role of CD4^+^ T cells but the population can be involved in DNA vaccine-mediated antitumor responses in which CD8^+^ populations did not play a central role [19]. We however demonstrated that the FasL DNA administration augmented production of anti-*β*-gal IgG Ab and IgG_2a_ Ab specific for a tumor antigen. Enhanced anti-*β*-gal Ab production suggested involvement of Ab-dependent cellular cytotoxicity that involved Ab binding to Fc receptors and/or complement-dependent cellular cytotoxicity that activated complement cascades. The previous study by Dharmapuri et al. also indicated that antitumor responses augmented by coinjected siRNA for Bak1 or caspase-8 were attributable to class switch from IgG_1_ to IgG_2a_ [18]. In fact, IgG_2a_ bound to Fc receptors better than other isotypes in a murine system [20]. In addition, comparison among immunoglobulin subtypes which, respectively, have a similar affinity to same antigen showed that IgG_2a_ activated complement greater than IgG_2b_ [21]. Nimal et al. showed increased T helper type 2 rather than T helper type 1 cell responses in vaccination with* FasL* gene-fused DNA and demonstrated that IgG_2a_ production was greater than IgG_1_ without generating T cell responses [22]. The data were concordant with the current study although their vaccination targets viral infections. The present data together with the previous studies collectively imply that expressed FasL at local DNA injection sites facilitated not only Ab production but also class switching, which resulted in augmentation of Ab-mediated cytotoxic reactions. Nevertheless, a precise mechanism of how the FasL molecules enhanced the humoral immunity is currently unknown. Cardiotoxin at the injection sites may also contribute to the humoral immunity since the treatment induces inflammatory reactions with local cytokine productions [14]. Proinflammatory cytokines such as interleukin-6, which is produced by cardiotoxin injection, potentiate B cell differentiation. Tissue destruction can therefore be crucial not only for integrating plasmid DNA but also for conditioning microenvironment for Ab production.

## 5. Conclusion

We demonstrated that administration of FasL DNA together with DNA encoding a putative tumor antigen gene produced antitumor effects on the antigen-expressing tumor cells* in vivo*. Cardiotoxin pretreatments enhanced expression of the DNA-encoded gene in muscle. The antitumor responses were not attributable to antigen-positive CD8^+^ T cells but associated with enhanced Ab production in particular IgG_2a_ subtype. The present study indicates a role of FasL DNA in augmentation of humoral immunity and suggests a potential application of FasL in DNA-mediated vaccine.
